# Combined Proteomics/Genomics Approach Reveals Proteomic Changes of Mature Virions as a Novel Poxvirus Adaptation Mechanism

**DOI:** 10.3390/v9110337

**Published:** 2017-11-10

**Authors:** Marica Grossegesse, Joerg Doellinger, Alona Tyshaieva, Lars Schaade, Andreas Nitsche

**Affiliations:** 1 Centre for Biological Threats and Special Pathogens, Highly Pathogenic Viruses (ZBS 1), Robert Koch Institute, Seestr. 10, 13353 Berlin, Germany; GrossegesseM@rki.de (M.G.); DoellingerJ@rki.de (J.D.); hellytish@gmail.com (A.T.); 2 Centre for Biological Threats and Special Pathogens, Proteomics and Spectroscopy (ZBS 6), Robert Koch Institute, Seestr. 10, 13353 Berlin, Germany; 3 Centre for Biological Threats and Special Pathogens (ZBS), Robert Koch Institute, Nordufer 20, 13353 Berlin, Germany; SchaadeL@rki.de

**Keywords:** poxvirus, adaptation, proteomics, genomics, cowpox virus, cell culture, passaging

## Abstract

DNA viruses, like poxviruses, possess a highly stable genome, suggesting that adaptation of virus particles to specific cell types is not restricted to genomic changes. Cowpox viruses are zoonotic poxviruses with an extraordinarily broad host range, demonstrating their adaptive potential in vivo. To elucidate adaptation mechanisms of poxviruses, we isolated cowpox virus particles from a rat and passaged them five times in a human and a rat cell line. Subsequently, we analyzed the proteome and genome of the non-passaged virions and each passage. While the overall viral genome sequence was stable during passaging, proteomics revealed multiple changes in the virion composition. Interestingly, an increased viral fitness in human cells was observed in the presence of increased immunomodulatory protein amounts. As the only minor variant with increasing frequency during passaging was located in a viral RNA polymerase subunit and, moreover, most minor variants were found in transcription-associated genes, protein amounts were presumably regulated at transcription level. This study is the first comparative proteome analysis of virus particles before and after cell culture propagation, revealing proteomic changes as a novel poxvirus adaptation mechanism.

## 1. Introduction

Cell culture propagation of viruses is a common prerequisite for investigating viral infection [1]. This is because only in rare cases the amount of virus particles isolated from a naturally occurring sample is sufficiently large for further characterization studies. Already in the very early days of studying viral infection in cell culture, an adaptation of the virus to the host cell was observed [2,3]. Numerous further reports can be found throughout the history of virus propagation in cell culture, underlining the importance of a comprehensive understanding of viral adaptation mechanisms [4,5,6,7].

While RNA virus adaptation mostly takes place by selection of genomic variants generated by erroneous replication, resulting in a less stable genome compared to DNA viruses [8], DNA virus adaptation mechanisms remain largely elusive. Low mutation rates of DNA viruses mainly result from the viral DNA-dependent polymerase 3′–5′ exonuclease activity, correcting for inaccurately incorporated nucleotides [9]. Additionally, some viruses, like herpes- [10] and poxviruses [11], encode proteins involved in DNA repair mechanisms. Hence, it can be hypothesized that DNA viruses may adapt via mechanisms other than de novo mutations and selection of genomic variants. Therefore, this study aimed to elucidate novel DNA virus adaptation mechanisms in cell culture by a combined proteomic/genomic approach, using poxviruses as a model.

As a member of the *Poxviridae* family, the genus Orthopoxvirus (OPV) comprises highly complex DNA viruses. With about 10^−8^ substitutions per nucleotide per replication cycle, OPV mutation rates are considered to be rather low [12]. Moreover, OPV encode at least four proteins involved in DNA repair mechanisms [11,13,14,15]. Nevertheless, besides their genome stability, OPV members like cowpox virus (CPXV) possess an exceptionally broad host range [16] and cause zoonotic infections [17], illustrating their adaptive potential in vivo. The number of zoonotic CPXV infections in Europe is increasing [18] and concerns have been raised recently about the emergence of variola virus-like viruses from zoonotic OPV, like CPXV and monkeypox virus, by natural evolution. This demonstrates the need for a comprehensive understanding of OPV adaptation [19] and encouraged us to choose CPXV as a model DNA virus for our proteomics/genomics approach.

Since isolated virus particles are capable of crossing the species barrier, it is assumed that adaptive changes can be identified in the virus particle itself. Copy number variation (CNV) has been suggested as the mechanism underlying rapid OPV adaptation [20]. However, CNV has only been described in cell culture applying high selective pressure [20,21,22]. But in naturally occurring OPV CNV has not been shown yet, possibly because it is a transient event [21]. Other OPV adaptation mechanisms include gene reduction [23] and recombination [24]. All these mechanisms describe changes in the viral genome. Although virions consist mainly of proteins, global proteomic changes in virus particles during cell culture passaging have not been analyzed to date.

We isolated CPXV mature virions from a rat crust and passaged them five times either in human epithelial cells (HEp-2), which are commonly used for virus propagation, or rat fibroblast cells (Rat-2), as rats represent a natural reservoir of CPXV. The appearance of the cytopathic effect (CPE) was cell-type specific but did not change among passages in the same cell line. However, an increase in virus yield was observed in the fifth passage in HEp-2 cells but not in Rat-2 cells. To elucidate mechanisms underlying this viral fitness change, we purified CPXV mature virus particles from the crust and each passage and analyzed the genome sequence by next-generation sequencing (NGS) and the proteome composition by tandem mass spectrometry (LC-MS/MS). Results showed that CPXV genomes were overall stable during passaging. In contrast, proteome analysis revealed numerous virus proteins that changed in an adaptation-related manner during passaging. While 15 and eight viral proteins changed during passaging in HEp-2 and Rat-2 cells, respectively, only a single viral protein changed comparably in both cell lines. Strikingly, proteins that increased in amount in HEp-2-passaged virions were mainly associated with viral immune evasion. These proteomic adaptations may explain the increase in viral fitness observed in HEp-2 cells. In search of mechanisms underlying the proteomic adaptation, we found a single minor variant increasing in frequency during passaging in both cell lines. Interestingly, the highest frequency (11.4%) correlated with the observed increase in viral fitness. The mutation was localized in the second largest subunit of the viral RNA polymerase *A25R* and led to an amino acid change from Lys to Thr. Besides protein kinase R (PKR) antagonism [21,22], *A25R* mutations are associated with transcript elongation in the literature [25,26,27,28]. Together with the fact that most minor genomic variants were located in transcription-associated genes, this suggests that proteomic virion changes are regulated on transcription level. Summarized, our results elucidate proteomic changes as a novel poxvirus adaptation mechanism which may also apply to other large nucleocytoplasmic DNA viruses.

## 2. Materials and Methods

### 2.1. Virus Isolation from Rat Crust

CPXV particles were purified from a crust originating from a Wistar rat which was intradermally infected with CPXV strain RatHei09/1 (NCBI GenBank: KC813504.1). Seven days post infection (p.i.) the crust was excised, cut into small pieces and homogenized with 20–25 ceramic beads in 500 µL of 10 mM Tris pH 9.0 at 6.0 m/s for 6 × 20 s using a FastPrep^®^ FP120 Homogenizer (MP Biomedicals, Santa Ana, CA, USA). After 3 min of sonication, the beads were pelleted and the supernatant was saved. The homogenized crust was washed twice with 500 µL of 10 mM Tris pH 9.0, saving the supernatants of the wash steps. To maximize virus yield, the remaining pellet was vortexed thrice in 500 µL of 10 mM Tris pH 9.0 for 30 s and washed again twice with 500 µL of 10 mM Tris pH 9.0. All supernatants, including those from washing steps, were pooled and nuclei and cell debris were pelleted at 5000× *g* and 4 °C for 5 min. The supernatant was sonicated for 1 min prior to mature virion purification by sucrose gradient centrifugation as described below.

### 2.2. Mature Virion Purification

Purification of intracellular mature virions (IMV) was done by rate-zonal sucrose gradient centrifugation [29] in sterile SW28 centrifuge tubes. For crude purification, the supernatant was centrifuged through a 36% sucrose cushion at 32,900× *g* and 4 °C for 80 min and the virus pellet was re-suspended in 1 mL of 10 mM Tris pH 9.0. The virus suspension was sonicated for 1 min, layered on a 24–40% continuous sucrose gradient and centrifuged at 26,000× *g* and 4 °C for 50 min. The virus band was collected and stored at 4 °C, while the pellet was re-suspended in 1 mL of 10 mM Tris pH 9.0, sonicated for 1 min and centrifuged through a fresh 24–40% continuous sucrose gradient as described before. The virus bands were pooled and concentrated at 32,900× *g* and 4 °C for 60 min. The pellet was re-suspended in 1 mL of 10 mM Tris pH 9.0 and stored at −80 °C.

### 2.3. Serial Passaging

For the analysis of CPXV adaptation in cell culture, purified virus particles from the crust were serially passaged in triplicate in HEp-2 (ATCC# CCL-23) and Rat-2 cells (ATCC# CRL-1764). Both cell lines were maintained in DMEM supplemented with 2 mM L-Gln, 5% FCS and 1% Pen/Strep. For infection of the first passage, 2 × 10^6^ cells in 4 mL of medium were infected with purified CPXV particles from the crust at an MOI of 0.1, assuming that the number of genome equivalents (GE) of gradient purified virions determined by qPCR equals the number of plaque forming units (PFU). Cells were incubated with daily CPE documentation for 4 days. Non-infected control cells served as a reference for CPE documentation. For virus isolation, cells were scraped into medium and pelleted at 1000× *g* and 4 °C for 5 min. The cell pellet was re-suspended in 1 mL of 10 mM Tris pH 9.0 and disrupted using glass beads. After pelleting of glass beads, viral DNA was extracted from the supernatant and the amount of GE was determined by qPCR. The next passage was infected at an MOI of 0.1, assuming a ratio of GE to PFU of 82:1 in HEp-2 and 17:1 in Rat-2 cells, as determined previously (Table S1). The according virus amount was added to the medium of an 80–90% confluent cell monolayer and incubated with daily CPE documentation for 4 days. The procedure of cell disruption, GE determination and infection of fresh cells was repeated 4 times for a total of 5 passages. Thereby, the number and size of cell culture flasks per replicate and passage was increased as follows: 1 × T25 cm^2^ (P1), 1 × T75 cm^2^ (P2), 5 × T175 cm^2^ (P3–5).

### 2.4. Plaque Assay and Determination of Genome Equivalents

The number of PFU and GE was determined by plaque assay and qPCR, respectively, as described previously [30].

### 2.5. Determination of the GE-to-PFU Ratio in the Supernatant

HEp-2 and Rat-2 cells in T25 cm^2^ cell culture flasks were infected in quadruplicate with CPXV strain RatHei09/1 at an MOI of 0.1. Four days p.i. cells were disrupted using glass beads and vigorous vortexing. Glass beads were pelleted and the supernatant was analyzed by plaque assay and qPCR.

### 2.6. Real-Time Cell Analysis

The xCELLigence RTCA (real-time cell analysis) system was used to compare the status of cells infected with the non-passaged crust and the passaged virions. Initially, background measurement was done with 50 µL of medium per well in a 96-well E-Plate. A total of 10,000 cells (HEp-2 or Rat-2) per well in 100 µL of medium were added and the Cell Index (CI) was monitored every 15 min. After overnight incubation, cells were infected with purified CPXV at an MOI of 0.1 in 50 µL of medium and impedance measurement was continued for 150 h [31].

### 2.7. Next-Generation Sequencing (NGS)

Viral DNA was isolated from purified mature virions using the PureLink^®^ Viral RNA/DNA Mini Kit (Invitrogen, Karlsruhe, Germany) according to manufacturer’s instructions but without carrier RNA. Libraries were prepared with the Nextera^®^ XT DNA Library Preparation Kit (Illumina, San Diego, CA, USA) according to the manufacturer’s instructions and sequenced using an Illumina MiSeq machine. Genomic sequence files are available from the NCBI Sequence Read Archive under the BioProject ID 399073.

### 2.8. NGS Data Analysis

Raw reads were de-multiplexed (CASAVA v1.8 from Illumina) and quality-trimmed (Trimmomatic v0.33 [32], FastQC v0.11.5 [33]). Fastq-files were received from the Bioinformatics Service Unit of the Robert Koch Institute. Separation of viral and host reads was done using RAMBO-K (Read Assignment Method Based On K-mers) [34]. Viral reads were de novo assembled using SPAdes v3.6.2 [35] and Velvet v1.2.09 [36]. Bowtie2 v2.2.6 [37] was used both for the confirmation of read separation by RAMBO-K and for the verification of de novo assemblies. Annotations were transferred from the CPXV GRI-90 reference strain (NCBI GenBank: X94355.2). As consensus sequences of replicates matched, reads of the triplicates per passage and cell line were summarized for further analysis. To identify genomic changes during CPXV passaging, assembled genomes were analyzed for variants/SNPs using the “find variations/SNPs” feature in Geneious v10.0.5 [38]. The minimum coverage was set to 100 with a minimum variant frequency of 0.01. Maximum variant *p*-value was 10^−6^ and minimum strand-bias *p*-value 10^−5^ when exceeding 65% bias. All consensus sequences containing variant annotations (11 sequences) were aligned using MAFFT high speed multiple sequence alignment program v1.3.5 with default parameters. Variant annotations were exported and manually reviewed. In the analysis, tandem repeats were defined as repetitive regions of at least one nucleotide with at least four repetitions. Mutations resulting in amino acid changes were added as isoforms to the proteomic database.

### 2.9. Sample Preparation for LC-MS/MS

Purified virus particles were pelleted in 500 µL of 10 mM Tris pH 9.0 at 25,000× *g* and 4 °C for 30 min. Virus pellets were lysed in 30 μL of lysis buffer (4% Sodium dodecyl sulfate (SDS), 10 mM Tris(2-carboxyethyl)phosphine (TCEP), 40 mM 2-Chloroacetamid (CAA) in 100 mM Tris pH 7.6) by heating at 95 °C for 5 min. Lysates were sonicated for 1 min, clarified at 16,000× *g* for 5 min and prepared for LC-MS/MS analysis using a modified Filter Aided Sample Preparation (FASP) method [39]. Briefly, 30 µL of lysate (<200 µg of protein, determined as in [30]) were filled up with 200 µL of 8M Urea in 100 mM Tris pH 8.5 (UA) and loaded onto a Microcon Centrifugal Filter Unit with 30 kDa molecular weight cut-off. SDS was removed by washing three times with 200 μL of UA. Urea was replaced by washing three times with 50 mM ammonium bicarbonate (ABC) and digestion was performed overnight at 37 °C in a wet chamber with Trypsin/Lys-C Mix in 40 µL of ABC using a protein: enzyme ratio of 25:1. Tryptic peptides were recovered by centrifugation and eluted twice with 40 μL of ABC. Peptides were desalted using 200 μL of StageTips with two Empore™ SPE Disks C18 [40], dried in a vacuum concentrator and stored at −80 °C until LC-MS/MS analysis.

### 2.10. LC-MS/MS Analysis

Single-run shotgun proteome analysis was performed using an EASY-nanoLC (Proxeon, Odense, Denmark) coupled to a linear trap quadrupole (LTQ) Orbitrap Discovery mass spectrometer using the parameters in Table S2. The sample order was shuffled during measurement to avoid systematic errors. The mass spectrometry proteomics data have been deposited to the ProteomeXchange Consortium via the PRoteomics IDEntifications (PRIDE) [41] partner repository with the dataset identifier PXD007567.

### 2.11. Proteomic Data Analysis

Quality control of proteomic data was routinely performed using the proteomics quality control (PTXQC) pipeline [42]. Identification and label-free quantification (LFQ) was done in MaxQuant v1.5.2.8, using the parameters in Table S3. Further bioinformatics analysis of protein and peptide identifications was done in Perseus. LFQ values were filtered for reverse hits, contaminants and proteins only identified by site. Log_2_ transformed protein intensities were separated in (i) crust + HEp-2 passages (18 samples) and (ii) crust + Rat-2 passages (18 samples). Each group was analyzed identically as follows: Rows were categorically annotated in crust (technical triplicates) and P1–P5 (biological triplicates each) and filtered for at least three valid values in at least one group. As linear correlation between crust and passaged virus was rather low (0.4–0.6 Pearson correlation), viral proteins were analyzed separately from host proteins. Missing values were imputed from normal distribution with default values mimicking low abundance measurements (width 0.3, down shift 1.8) and median column normalization was performed to account for different sample loads. Principal component analysis was done prior to z-score normalization. Significant differences of z-score normalized protein intensities were analyzed using an analysis of variance (ANOVA) test with 5% permutation-based false discovery rate (FDR) and 250 randomizations. ANOVA significant protein intensities were averaged and courses were correlated to hypothesized adaptation-specific reference profiles, which were generated using the reference profile feature included in the Perseus profile plot analysis (Figure S1). Proteins with a distance ≤0.1 to the reference profiles were defined as being adaptation specific. Identical viral proteins correlating with the same reference profiles in HEp-2 and Rat-2-passaged cells were defined as comparable between cell lines. Host proteins were analyzed analogously to viral proteins. The crust was excluded from the analysis because of the low linear correlation compared to cell culture-passaged virus. LFQ values were filtered additionally for rat proteins in HEp-2 samples and vice versa to remove host-unspecific proteins. Rows were filtered for at least 12 valid values and an ANOVA test was performed applying 1% permutation-based FDR with 2500 randomizations. Mean values of ANOVA significant proteins were hierarchically clustered using Euclidean distance and 300 clusters. Protein clusters including proteins increasing or decreasing during passaging were defined as adaptation-specific host proteins.

## 3. Results

### 3.1. Replication Gain during Passaging in Human Cells

**Figure 1:**
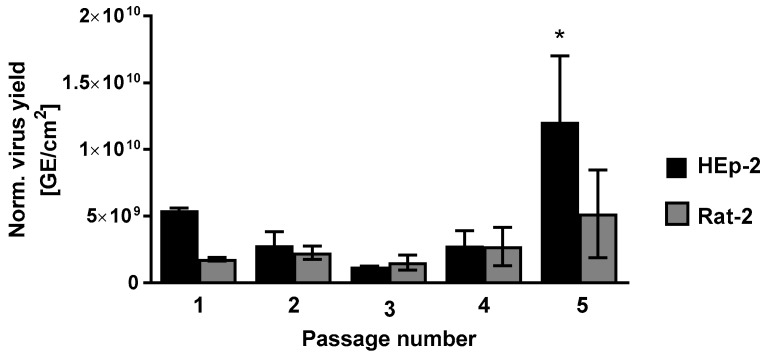
Virus yield during CPXV crust passaging reveals a replication gain in human cells. Viral genome equivalents (GE) of passaged CPXV crust were determined by qPCR after cell lysis. Shown are mean values ± standard deviation (SD) of normalized virus yield. Statistics: One-way analysis of variance and Bonferroni’s multiple comparisons test (* *p* ≤ 0.05).

CPXV IMV particles were isolated from a rat crust and serially passaged five times in triplicate in HEp-2 and Rat-2 cells. To estimate changes in viral fitness during passaging, the CPE was documented and the virus yield determined. A cell type-specific CPE was detectable in both cell lines in all passages. While Rat-2 cells showed a CPE on the first day p.i., HEp-2 cells showed morphological changes on the third day p.i. However, morphological changes remained the same during passaging in both cell lines (Figure S2). Although HEp-2 and Rat-2 cells showed diverse CPE appearance, the total virus yield in both cell lines was comparable, except for the fifth passage showing higher virus yields in HEp-2 cells. In contrast, virus yields in Rat-2 cells showed no significant differences during passaging. This indicated a replication gain of CPXV in human cells but not in rat cells (Figure 1).

### 3.2. Real-Time Cell Analysis Indicates Heterogeneities of the Crust Isolate

**Figure 2:**
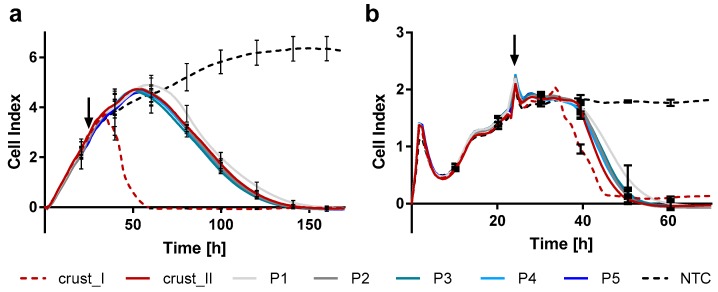
Real-time cell analysis of HEp-2 and Rat-2 cells infected with CPXV crust and passages reveals no differences among passages but heterogeneities of the crust. (**a**) HEp-2 and (**b**) Rat-2 cells were seeded in 96-well E-plates and cell adherence was measured over time. About 24 h after seeding, cells were infected with purified CPXV originating from a rat crust or from different passages (P1–P5) at an MOI of 0.1 (arrow). Shown are mean values ± SD of biological triplicates of passages measured in technical quadruplicates (*n* = 12) or six technical replicates of the crust, which cluster in two different courses (crust_I and II) with each *n* = 3. NTC: non-infected cells.

Infection kinetics of CPXV crust and passages were studied using RTCA which is based on impedance measurement by electrodes embedded in the well bottom. The cell index (CI) represents the background-corrected relative impedance and increases with increasing interaction of cells with the well bottom and vice versa. Therefore, the CI depends on cell status parameters like cell number, morphology and adhesion. Passaged CPXV were analyzed with the same cells they had been propagated in previously, revealing an overall faster detachment of Rat-2 cells compared to HEp-2 cells. Passages showed comparable homogenous courses in both cell lines. In contrast, replicates of the crust showed clearly heterogeneous courses which clustered in two distinct courses: one representing the course of the virus passages and another showing significantly faster cell detachment (Figure 2). These observations were confirmed on repetition of the experiment (Figure S3). Since purified virus particles were used, toxic effects may be excluded. Instead, heterogeneous courses may indicate that the crust contained a heterogeneous virus population.

### 3.3. Genome Analysis of CPXV Crust and Passages

In the following, samples are abbreviated according to cell line and passage, e.g., “RP1” for first passage in Rat-2 cells. To elucidate possible genomic changes underlying an increased viral fitness in HP5, and moreover to elucidate possible changes in virus particles occurring in the absence of CPE and yield changes, we applied deep sequencing to the crust and each passage. We obtained mean genome coverages of more than 2500 in passages and about 1300 in the crust (Table S4). De novo assembled viral genomes had a length of >216,300 bp, except for RP1 with 209,458 bp. In comparison, the genome size of the CPXV strain RatHei09/1 used is specified with 208,980 bp in the NCBI database (GenBank: KC813504.1). All 206 unique coding sequences (CDS) in the GRI-90 reference strain could be transferred to assembled genomes, except for RP1. Here sequence information of the terminal five genes was missing. Note that the terminal five CPXV genes D1L–D5L are inverted versions of I1R–I5R located in the inverted terminal repeat region.

De novo assembled viral genome sequences did not reveal any variation in copy number. Assemblies were verified by alignment of reads to the genome sequence. In doing so, duplicated genes showed a clearly higher coverage than genes with a single copy, as demonstrated by the terminal five genes with known duplication (Figure S4). Coverage of these genes was consistently higher than the mean coverage across the genome adding two standard deviations. However, no other gene with comparable increased read depth was identified in any of the samples, indicating that CNV did not occur during passaging of the CPXV crust isolate.

#### Variant Analysis Reveals Only Minor Variants during Passaging

Comparison of consensus sequences revealed six differences between crust and passages. All six variants were insertions or deletions localized in repetitive sequences outside of CDS. Notably, HP5 and RP5 consensus sequences differed from the crust only by 2 and 4 nt, implying the overall genome stability of CPXV during a 5-passage cell culture propagation. Nevertheless, variant analysis revealed at least 23 minor variants with more than 1% frequency in each sample. Notably, about 72% of all variants were localized in tandem repeats. Among a total of 78 different variants in HEp-2 and 89 in Rat-2 cells, 54 were identical in both cell lines, showing also reproducible variant frequencies. But despite these correlations, mutations shared in both cell lines were for the most part not detected in the same passage, indicating a common underlying mechanism, which may temporally differ between cell lines (Figure S5).

Altogether 14 mutated CDS were identified, including 12 CDS with amino acid change (Table 1). Surprisingly, half of these CDS were essential genes associated mostly with transcription. The term de novo mutation is used in the following to describe variants appearing for the first time in a passage. De novo mutations were identified in both cell lines in the *A26L* gene encoding the A-type inclusion protein, the uncharacterized *D6L* gene and the *E10R* gene encoding an mRNA-de-capping protein (Table 1). Additionally, *A26L*, *E10R* and the *A25R* gene encoding the RNA polymerase 133 kDa polypeptide contained variants which were also detected in the crust, indicating pre-existing variants rather than de novo mutations. Furthermore, a frequency accumulation of a single variant was observed. This was an A→C transversion at position 269 in *A25R* leading to a K→T substitution (Lys90Thr). While variant frequency of this mutation in the crust was 3.1%, HP5 displayed a frequency of 11.4% and RP4 a frequency of 9.5%. The Lys90Thr in the *A25R* gene was the only CDS-localized mutation detected in every sample and, moreover, the only mutation that increased in frequency during passaging, indicating a selective advantage of this mutation.

As HP5 was the only sample showing a significant increase in virus yield, mutations that were exclusively found in this passage may be relevant. Mutations limited to HP5 were identified in the *E10R* gene, the uncharacterized *C1L* gene and the *H3R* gene, encoding a late transcription elongation factor. However, variant frequencies were rather low (<1.8%).

### 3.4. Proteome Analysis of CPXV Crust and Passages

The proteome composition of non-passaged and passaged CPXV IMV particles was analyzed by LC-MS/MS to elucidate possible proteomic changes. A total of 167 viral proteins and 3286 host proteins (human, rat or contaminant) were identified. It should be noted that proteins outside of virions may stick together with virion proteins and hence cannot be entirely removed during purification, meaning that identified proteins may be incorporated in or associated with IMV particles.

#### 3.4.1. Changes of Viral Proteins during Passaging

Mutations resulting in amino acid changes in viral proteins were included in the viral protein database used for proteome analysis. Not surprisingly, as mutation frequencies were low (≤11.4%), no isoforms were identified in proteome analysis.

**Figure 3:**
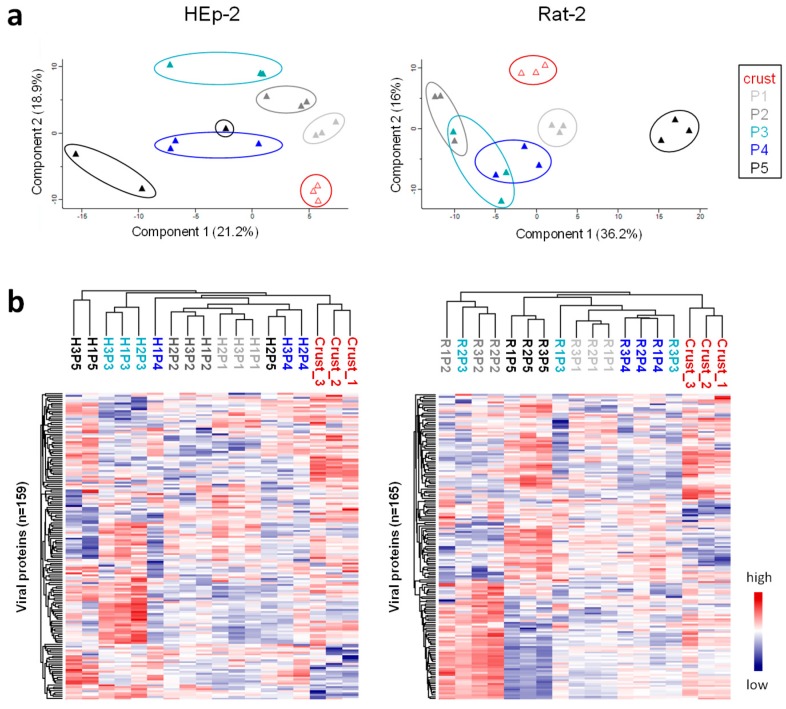
Principal component analysis and hierarchical clustering of CPXV IMV proteins reveal proteomic differences among passages. Normalized label-free quantification (LFQ) intensities of virus proteins without missing values were used for (**a**) principal component analysis prior to z-score normalization and (**b**) hierarchical clustering after z-score normalization.

A total of 167 highly confident CPXV proteins were identified. Proteins quantified in all triplicates in at least one sample were considered for further analysis. Because linear correlation between crust and cell culture passages was rather low, as demonstrated by a Pearson correlation of 0.4–0.6, host proteins were removed from the analysis and analyzed separately, improving linear correlation of virus proteins to >0.8 and even >0.9 between replicates (Figure S6). This indicated great differences of host proteins in crust and passaged virus preparations, as expected for virus particles isolated from diverse matrices like rat skin and cell culture monolayer. Surprisingly, principal component analysisand hierarchical clustering of virus proteins revealed fairly good separability of CPXV particles isolated from different passages (Figure 3). This suggested that the IMV proteome changed during cell culture passaging, which is underlined by the heat map visualization in Figure 3 showing considerable differences between crust and passages and also differences among passages.

**Figure 4:**
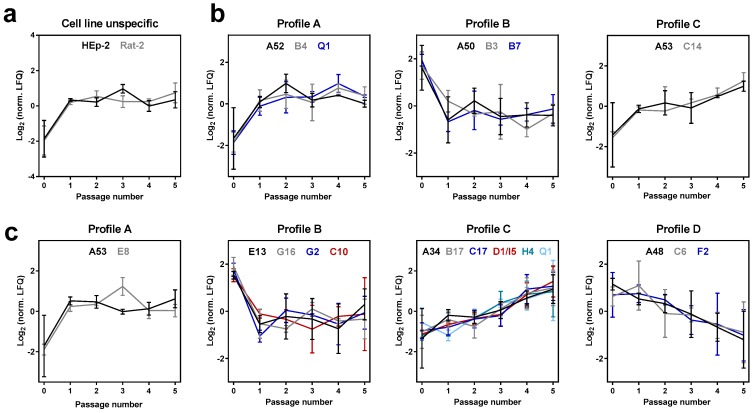
Viral proteins changing in an adaptation-associated manner during passaging in HEp-2 and Rat 2 cells. Proteins are sorted according to adaptation-associated time courses with distances ≤0.1 to reference profiles A–D, which are shown in Figure S1. (**a**) A single viral protein (G13) displays comparable courses during passaging in both cell lines (cell line unspecific). Proteins changing in a cell-line-independent manner were identified in (**b**) Rat-2- and (**c**) HEp-2-passaged virions. Shown are normalized LFQ intensities (log_2_) of ANOVA-significant proteins. Error bars indicate means ± SD of three biological replicates (passages 1–5) or three technical replicates (passage 0: crust).

Significance of differences was analyzed by ANOVA and adaptation-related viral proteins were defined as proteins displaying a clear abundance trend during passaging or showing altered amounts upon host change, meaning different amounts in crust and passages. To identify common adaptation mechanisms, courses of viral proteins were compared between cell lines. This led to the identification of the envelope protein G13 which changed comparably during passaging in both cell lines (Figure 4a). Proteins with different courses in cell lines during passaging were considered to be cell line specific, resulting in 15 viral proteins in HEp-2-passaged virions and eight viral proteins in Rat-2-passaged virions (Figure 4b,c). The courses of these proteins revealed that the same number of viral proteins changed upon infection of the first passage (six proteins per cell line; Figure 4b,c), while more viral proteins changed during passaging in HEp-2 cells (nine proteins; Figure 4c) compared to Rat-2 cells (two proteins; Figure 4b). Moreover, two proteins (A53 and Q1) changed in virions passaged in both cell lines but showed different adaptation-associated courses (Figure 4b,c).

Viral proteins that changed in a cell-type-specific manner had similar functions in both cell lines, including proteins associated with viral membrane, immune evasion and DNA replication as well as proteins with unknown function (Table 2). Membrane-associated proteins changed upon the first passage in cell culture (E8 and E13 in HEp-2-passaged virions; B4 in Rat-2-passaged virions). An exception was the membrane glycoprotein A34, which continuously increased in amount during passaging in HEp-2 cells. Notably, all four immunomodulatory proteins (B17, C17, D1/I5 and Q1) that changed in HEp-2-passaged virions increased continuously in amount during passaging. In contrast, the two immunomodulatory proteins (B7 and Q1) that showed altered protein amounts in Rat-2-passaged virions changed upon the first passage but remained constant during further passaging (Figure 4 and Table 2). Moreover, virions passaged in HEp-2 cells showed changes in viral proteins associated with diverse other processes, including viral proteins with enzymatic activities (A48, G2 and H4).

#### 3.4.2. Changes of Host Proteins during Passaging

In contrast to the passaged samples, the crust host proteome was dominated by rat collagen-α 1 and 2, which is the predominant fibril-building collagen type of the skin. It was no surprise to identify this protein in extraordinarily high amounts in a sample originating from rat skin. However, for the analysis of changes in host protein abundance, the crust had to be excluded, because similarity to the passaged virion host background was too low for comparison, as indicated by a poor Pearson correlation (<0.5).

Except for P3 and P4 in Rat-2 cells, all passages could be separated by principal component analysis, indicating differences in host cell protein abundance during passaging (Figure S7). The ANOVA test revealed 122 human proteins significantly altered in HEp-2-passaged virions and 398 rat proteins in Rat-2-passaged virions. Mean values of ANOVA-significant proteins were analyzed for adaptation-related courses using Perseus. Because the crust had to be excluded from the analysis, host proteins with different amounts in the crust and in passages could not be identified. Although most host proteins did not show any adaptation-related courses, host proteins with changing amounts during passaging were identified (Figure S8). Because LC-MS/MS is a highly sensitive analytical approach, it is clear that many contaminating proteins are identified, as demonstrated by 3453 identified host proteins. To discriminate between contaminant and virion-associated host proteins, an enrichment analysis may be done, comparing the amount of host proteins in virions to the host protein expression in cells [30]. Although this was not feasible in the present study, because no reference values of HEp-2 and Rat-2 protein expression were available, most host proteins that changed during passaging in an adaptation-related manner have been previously identified in highly pure OPV IMV preparations, as indicated in Figure S8 [30]. Enrichment values of proteins previously characterized as virion associated are used in this study to discriminate between virion-associated host proteins and contaminants [30]. Thereby, proteins with at least a two-fold enrichment in OPV IMV particles compared to the whole cell were considered as virion associated, resulting in twelve and eight host proteins in HEp-2- and Rat-2-passaged virions, respectively (Table 3). Only the translation initiation factor 3 subunit B (eIF3B) changed in virions passaged in both cell lines, showing increasing abundance during passaging. Interestingly, a protein associated with cytoplasmic DNA sensing (XRCC5) increased and an apoptosis-associated protein (PDCD6) decreased in abundance in HEp-2-passaged virions. In contrast, in Rat-2-passaged virions a serin/threonine-protein phosphatase increased in abundance (Ppp2r4), while another one (Ppp2r1a) decreased during passaging. Moreover, a regulatory subunit of the proteasome (Psmc5) increased during passaging in Rat-2 cells, while the proteasome activator complex subunit 4 (Psme4) decreased in abundance.

## 4. Discussion

Cell culture passaging is the gold standard for virus isolation and propagation [1]. Vaccinia virus (VACV), the OPV member used as a vaccine in smallpox eradication, was the first virus grown in vitro [43] and its adaptation to cell culture has already been observed in the 1960s, reporting initially poor virus growth that improved during passaging [3,44]. At that time the observed adaptation was thought to result from a selection of pre-existing genomic variants [44]. However, by now it is known that this is only one possible adaptation mechanism of poxviruses. There are diverse studies verifying the adaptive potential of poxviruses in cell culture on the genomic level. However, these studies used already cell culture-adapted viruses like VACV [45], extreme cell culture passaging, e.g., more than 500 passages for modified vaccinia virus Ankara (MVA) [46], or artificial selection pressure on distinct genes by knockdown of complementary genes [20]. Therefore, prior to this study, it remained elusive how poxvirus particles adapt to cell culture right after isolation from a natural host without selective pressure by gene knockout. As for research purposes, commonly less than five passages are sufficient to isolate and propagate OPV for further analyses, these first passages are of special interest. It is known that extensive cell culture passaging leads to altered or even attenuated viruses, like MVA [46], but changes of virus particles during early passages have been hardly investigated prior to the present study.

In this study, modern omics-techniques were applied to analyze the adaptation of CPXV in cell culture during the first five passages. Poxvirus adaptation in cell culture may be primarily observed by differences in CPE or virus yield. These changes may be detected by standard methods, like plaque assay or qPCR, which are generally performed during virus isolation in cell culture. In the present study, CPXV particles were isolated from a rat crust and passaged five times in a human and a rat cell line. Passaging resulted in reproducible viral fitness between cell lines during the first four passages. However, an increase in viral fitness was observed in human cells in the fifth passage, indicating an adaptation of CPXV to human cells. As rat cells are closer related to the host organism from which CPXV were isolated than human cells, a lesser degree of adaptation to these cells seems plausible. Nevertheless, passaged viruses showed no differences in RTCA in both cell lines. As RTCA was based on measurement of cell attachment to the surface, it seems conclusive that results are in agreement with CPE observation which revealed no differences.

Genomic adaptation of CPXV may occur either by CNV [20], de novo mutations or selection of pre-existing variants. In the present study CNV was not detected and the overall CPXV genome stability during passaging in two different cell lines was shown. Although no structural variant became dominant during passaging, deep sequencing allowed the identification of minor variants in each passage. Strikingly, variants were primarily found in tandem repeats. These so-called microsatellites can be found throughout the whole poxvirus genome, accounting for about 24% of the sequence [47]. Previously, early stop mutations were found to be accumulated in chordopoxvirus microsatellites, leading to the hypothesis of microsatellite hypervariability as a major source of poxvirus genome variability and hence as a source of poxvirus adaptation [47]. Our data suggests that hypervariability in microsatellites is a CPXV adaptation mechanism in cell culture.

CDS-localized mutations were primarily found in transcription-related genes. This seems conclusive, because poxviruses replicate in the cytoplasm of the host cell and therefore need to encode their own transcription machinery which may adapt to the host. However, variant frequencies of transcription-associated genes were rather low (≤3%), except for a single substitution in the *A25R* gene, showing up to 11.4% variant frequency in virions passaged in human cells. This Lys90Thr substitution in the RNA polymerase gene *A25R* was the only variant accumulating in frequency during passaging. The OPV DNA-directed RNA polymerase is composed of eight subunits, which partially display similarity to the eukaryotic polymerase [48,49]. Also the second largest subunit A25 displays homology to the eukaryotic RNA polymerase [50]. Mutations in the *A25R* gene during OPV cell culture passaging have already been described in the literature [21,22,25]. Most *A25R* mutations are associated with isatin-β-thiosemicarbazone (IBT) resistance or reduced IBT sensitivity [22,25,26,27]. IBT treatment leads to an abortive infection, presumably by increasing RNA processivity by either inhibiting transcription termination or stimulating elongation, but the detailed mechanism is still unknown [22,28]. Cone [21] and also Brennan and colleagues [22] observed *A25R* mutations in cell culture while applying selective pressure to different PKR inhibitors. PKR is an IFN-stimulated gene and functions as antiviral sensor protein of the host cell which detects viral dsRNA, leading to phosphorylation of the eukaryotic translation initiation factor *eIF2α*, which in turn leads to translational shutdown. Established CPXV PKR antagonists are encoded by the *M3L* and *F3L* genes [51,52]. It has been reported that passaging of an *F3L* knockout mutant leads to gene expansion of *M3L*, which goes along with an adaptive mutation in the *A25R* gene. Although this mutation reduced the dsRNA level, it also activated PKR [21]. Furthermore, it has been shown that introduction of a rhesus cytomegalovirus-derived PKR antagonist (*rhtrs1*) in an *M3L*/*F3L* double-knockout strain leads to either gene expansion of *rhtrs1* or to mutations in the *A25R* or *A36R* gene, both of which improving replication during cell culture passaging [22]. Summarized, the literature data suggests that mutations in the *A25R* gene are related to transcription elongation and PKR antagonism [28]. Hence, the accumulation of the Lys90Thr variant during passaging of a CPXV crust isolate in two different cell lines may be related to altered transcription, although the mutation site does not agree with mutations reported in the literature [21,22,25,27]. Nevertheless, it can be stated that the second-largest subunit of the OPV RNA polymerase is a major target of adaptive mutations. Furthermore, it may be speculated whether the Lys90Thr variant observed at the highest frequency in the fifth passage in human cells may be responsible for the significant increase in virus yield in this passage. One may argue that this mutation was also observed in rat cell-passaged virions which did not show increased virus yield. But frequency in rat cell-passaged virions was lower and effects may also be host dependent. Additionally, another mutation in a gene associated with transcription elongation was identified exclusively in the fifth passage in human cells. This gene encodes the positive late transcription elongation factor H3 [53]. Therefore, the increase in virus yield may also be a combination of *A25R* and *H3R* mutations.

Apart from minor variants, CPXV genomes were remarkably stable during passaging, suggesting adaptation on a different level. Hence, we analyzed global proteome changes of CPXV IMV particles during passaging. Not only viral proteins that changed upon switch from the crust to the first passage in cell culture but also changes in viral and host proteins during passaging were identified. For the first time, this data proves that poxvirus adaptation takes place on virion proteome level. However, it should be noted that the present study is descriptive and, from the observation of proteomic changes in the presence of viral fitness changes, an association between both was hypothesized. Viral proteins displaying adaptation-associated changes can be functionally categorized in proteins associated with membrane, immune evasion and proteins with diverse enzymatic activities, as discussed in the following.

OPV enter the host cell either by fusion with the plasma membrane or internalization by endocytosis [54]. OPV entry has been shown to differ in a species- and strain-specific manner and cell culture passaging has been suggested to contribute to the entry adaptation process [54,55,56]. During CPXV crust passaging in different cell lines, the abundance of the viral protein G13 changed in a cell line-independent manner. G13 is the most abundant viral envelope component in extracellular enveloped virion (EEV) particles and target of the anti-poxvirus drug ST-246 [57]. Although purified IMV particles were analyzed and the co-purification of EEV particles seemed unlikely because of their unstable outer membrane [58], it has been shown that EEV proteins can be detected even in highly pure CPXV IMV preparations [30]. G13 plays a role in wrapping of IMV to produce EEV particles [57] and G13-knockout mutants lead to severe attenuation in mice [59]. In cell culture, deletion of G13 has been shown to result in smaller plaque size [59]. However, the role of increased G13 amounts in IMV particles passaged in HEp-2 and Rat-2 cells remains elusive. Furthermore, changes in A34 and B4 protein amounts were detected during cell culture passaging in human and rat cells, respectively. A34 in combination with A37 is required to repulse superinfecting virions. The A34/A37 complex localizes on the surface of infected cells and induces actin tail formation upon contact with new virions containing the B4 protein, leading to repulsion of virions to uninfected cells [60,61]. This mechanism allows a four times faster virus spread than the replication cycle would permit. Moreover, an early expression of A34 and A37 is required for effective virus spread and normal plaque formation in cell culture [60]. Increased amounts of B4 and A34 in passaged virions may indicate an enhanced capacity for repulsing virions from already infected cells. Incorporation of these proteins in virus particles would enable a repulsion of virions right after infection, even before early viral protein expression. This hypothesis may be underlined by the observation of an increased plaque size during passaging (Figure S9). However, as an increase in plaque size was observed independently of the cell line, it may be hypothesized that increased virus spread is a general mechanism underlying CPXV adaptation in cell culture.

Poxviruses are remarkably complex viruses which encode diverse proteins to evade the host immune system. Interestingly, all immunomodulatory proteins that increased in virions during passaging (B17, C17, D1/I5 and Q1) are virulence factors in vivo [62,63,64,65]. B17 is a soluble IFN receptor which possesses a remarkably broad capacity of binding and inhibiting type I IFN from different species, e.g., human, rat, mouse, bovine and rabbit [66]. While VACV encoding B17 is able to suppress IFN-α in mice, infection with a B17-deficient strain induces a type I interferon antiviral response [67]. Replication of a VACV B17 knockout mutant is reduced in cell culture in the presence of IFN, demonstrating that B17 is also advantageous for OPV replication in vitro [68]. Inflammation may be induced by the complement cascade or pro-inflammatory cytokines like CC chemokines. C17 is a complement-binding protein, interfering with innate and adaptive immune system components [65,69]. The D1/I5 protein is a chemokine-binding protein which is secreted from infected cells to inhibit CC chemokine-mediated immune cell activation [70]. Q1 is the only immunomodulatory protein that changed during passaging in both cell lines. While in Rat-2-passaged virions Q1 amounts increased upon the first passage, Q1 amounts were continuously increasing during passaging in HEp-2 cells. The CPXV Q1 protein inhibits NF-κB and IRF-3 activation, although the mechanism remains unclear [71,72]. Moreover, pro-inflammatory cytokine secretion from primary monocytes is inhibited in the presence of Q1 [73]. However, the role of B17, C17, D1/I5 and Q1 in cell culture remains to be elucidated. Nevertheless strikingly, amounts of these proteins continuously increased in virions passaged in human cells. Hence, it may be hypothesized that changes in the amount of immunomodulatory proteins, which are most abundant in the fifth passage, may underlie the increased viral fitness observed in this passage in human cells.

Besides proteins of unknown function and viral proteins associated with membrane and immune evasion, proteins associated with other enzymatic activities changed in cell culture-passaged virions, including A48, A53, G2 and H4. The A48 protein encodes a protein with partial sequence homology to the superoxide dismutase. But A48 does not show enzymatic superoxide dismutase activity, because it lacks residues which are important for the according catalytic activity. As A48 influences neither replication nor virulence [74], the role of this protein during poxvirus cell culture adaptation remains elusive. The viral DNA ligase A53 increased in amount upon passaging in human cells and during passaging in rat cells a continuous increase of A53 was observed. A53 recruits a host factor, the cellular topoisomerase II, to viral replication sites during infection [75]. However, topoisomerase II amounts did not change during passaging. The G2 protein is a dUTPase involved in DNA repair, minimizing dUTP incorporation in viral DNA [76]. Upon passaging in human cells, G2 amounts decreased in virions, coinciding with the fact that G2 deletion does not impact replication in vitro [76]. Finally, amounts of the oxidoreductase H4 increased continuously during passaging in HEp-2 cells. Poxviruses encode their own disulfide bond formation machinery which is composed of the F10, the A3 and the H4 protein. H4, also known as poxvirus glutaredoxin-2, is the last protein among these three enzymes which act in a cascade-like manner [77]. H4 is known to catalyze the disulfide bonds in viral membrane proteins and, moreover, is suspected to catalyze disulfide bonds in core and lateral body proteins [78]. Poxviruses encode two glutaredoxin proteins, H4 and R2. While R2 is non-essential for virus replication, H4 plays an essential role during virion assembly [79,80]. Hence, it may be speculated whether increased amounts of H4 may be linked to enhanced viral fitness in the fifth passage in HEp-2 cells. However, there is no literature available supporting this hypothesis.

Generally, changes in the amount of viral proteins during cell culture propagation may result from either altered amounts of these proteins, higher incorporation rates in virus particles or a combination of both. Altered protein amounts may be regulated on transcription or translation level or by protein stability. Poxviruses encode their own transcription machinery [81]. As genomic changes were mainly detected in transcription-related genes, it may be hypothesized that proteomic changes are regulated on the transcription level.

The incorporation of host proteins in virions is a possible adaptation mechanism. VACV EEV particles have been reported to incorporate host complement control proteins [45,82], cytoskeletal proteins and chaperones [83]. Host protein expression is nearly shut down during poxvirus infection by shutting down host mRNA expression [84]. Nevertheless, some host proteins remain constantly expressed or even differentially regulated during infection, e.g., the mitochondrial proteins ND4 and COX-2 and heat shock protein HSP90 [85]. Additionally, host proteins are recruited to virus factories during infection, e.g., topoisomerase II [75], ubiquitin [86] and the translation initiation complex [85]. Moreover, transcription of poxvirus intermediate and late viral genes depends on host factors. Host G3BP and p137 (CAPRIN-1) are involved in intermediate transcription [87], while hnRNP A2/B1 [88] and RBM3 [89] are known to play a role in late viral promoter activation. All of these factors, except for ND4 and COX-2, were identified in the present study. However, only eIF3B changed in an adaptation-associated manner and was considered to be virionassociated [30]. Moreover, eIF3B is the only host protein that changed in both cell lines, showing increasing amounts during passaging. It is the RNA-binding component of the eukaryotic translation initiation factor 3 and part of the translation initiation complex, which is recruited to virus factories during OPV infection [85]. Hence, it seems plausible that eIF3B may be incorporated in IMV particles during assembly but its role for CPXV adaptation remains elusive. Apart from this single host protein changing during passaging in both cell lines, eleven proteins changed exclusively in HEp-2-passaged virions and seven in Rat-2-passaged virions.

Virion-incorporated cellular Profilin decreased during passaging in HEp-2 cells. Poxviruses encode a viral Profilin protein A44 but in contrast to cellular Profilin, which modulates actin dynamics, poxvirus Profilin rather influences polyphosphoinositides metabolism [90]. One may speculate that viral Profilin amounts may increase, since cellular Profilin amounts decrease. However, this was not the case. ARF1 amounts decreased during passaging in HEp-2 cells. ARF1 is involved in the formation of Golgi-coated vesicles. During poxvirus infection, IMV particles are wrapped with trans-Golgi membranes to form IEV particles but the role of ARF1 in poxvirus infection is unknown [91]. Moreover, PDCD6 amounts decreased during passaging in HEp-2 cells. PDCD6 is involved in endoplasmic reticulum-Golgi vesicular transport but has not been associated with a role during poxvirus infection. XRCC5, also known as Ku80, is part of a large cellular DNA repair complex which functions as pattern recognition receptor for DNA viruses, activating innate immune response. Poxviruses encode a Ku80 antagonistic protein (D5L/I1R) which blocks binding of the Ku80 complex to DNA [92]. Although it appears conclusive to identify Ku80 in poxvirus mature virions, increased amounts during passaging in human cells cannot be explained. Host proteins changing in Rat-2-passaged virions have not been directly linked to poxvirus infection. However, interestingly, a regulatory subunit of the proteasome (Psmc5) increased in abundance during passaging. Although multiple proteasomal subunits have been identified in highly pure OPV preparations [30], the localization of the proteasome during poxvirus infection has not been analyzed yet. For example, it has been reported that proteasomal subunits are recruited to replication centers during cytomegalovirus infection [93].

Although multiple adaptation-related changes of virus and host proteins were identified during passaging, general and technical limitations of the present study should be considered. We analyzed the proteomic composition of virions and, in contrast to the viral genome, the proteomic stability of OPV remains elusive. It has been stated in the most extensive OPV proteome study to date that the virion proteome composition cannot be determined exactly by a fixed set of proteins [30]. Particularly, the relevance of the identified proteins and, moreover, the correlation of the virion proteome and the phenotype are not known. Identified proteins may not get into the host cell or the observed protein changes may be even too low to induce any phenotypic effect. Therefore, the biological significance of identified proteins as well as the correlation between different virion protein amounts and viral fitness have to be analyzed in more detail in the future. A technical limitation of the applied shotgun proteome analysis is that the absence of a protein cannot be stated by the experimental design. Peptides were analyzed in a data-dependent manner, meaning that the most intense peptides are selected for fragmentation. This in turn means that peptides may be not identified because they are absent, or because they are present at low abundance and hence not subjected to fragmentation.

The new insights given by the present descriptive study may be used for further investigations. The detected changes of protein amounts associated with virions indicate a regulatory mechanism: Either a mechanism for the selective protein incorporation in virions or a non-selective incorporation as a result of a general regulation of protein amounts during infection. It is very interesting how OPV accomplish this regulation. Our data suggests that transcriptional regulation plays a role in this context. The effect of the Lys90Thr mutation in the viral RNA polymerase subunit *A25R* on transcription and protein level seems particularly interesting for detailed analysis.

## 5. Conclusions

This study demonstrates that DNA virus particles change during passaging in cell culture, even at a low number of passages. These changes may be detected by changes in viral fitness but they may be also “silent”, meaning that virion changes do not result in viral fitness changes. We show that virion proteome changes may be associated with changes in viral fitness upon host switch, which was simulated by a cell line species different from the natural host organism. In contrast, proteomic changes without viral fitness changes were observed during passaging in a cell line originating from the host species from which the virus was isolated. Our data reveal that proteomic adaptation presumably contributes to virus adaptation to new hosts, allowing a more rapid adaptation of viruses with a stable genome, namely DNA viruses. Finally, DNA virus adaptation in cell culture, in the absence of major genomic variant changes and, moreover, in the absence of viral fitness changes, should be kept in mind when working with cell culture-propagated DNA viruses, especially nucleocytoplasmic large DNA viruses like poxviruses.

## Figures and Tables

**Table 1:** Mutations in coding sequences during CPXV crust passaging.

CDS ^a^	Pos. ^b^	Nucleotide Change	PE ^c^	Amino Acid Change		Variant Frequency HEp-2					Variant Frequency Rat-2				
					P0	P1	P2	P3	P4	P5	P1	P2	P3	P4	P5
*A12R*	728	A→G	S	K→R										1.2	
*A24R*	1149	(A)_8_→(A)_9_	FS	none										1.4	
*A25R*	269	A→C	S	K→T	3.1	2.7	4.2	6.4	2.9	11.4	2.2	4.2	2.7	9.5	4.6
*A25R*	4	(A)_8_→(A)_9_	FS		1.3									1.4	
*A26L*	1091	G→T	S	H→Q	1.1										
*A26L*	1118	(GTT)_8_→(GTT)_9_	I	P→QP	3.1	2.7	2.9		2.6	3.4		2.6		4.2	2.5
*A26L*	1115	(GTT)_8_→(GTT)_7_	D	QP→P	4.6	4.3	3.8		3.8	4.2		4.6			
*A26L*	1093	(TTG)_8_→(TTG)_7_	D	Q→				3.7			5.0		3.4		
*A26L*	1093	(TTG)_8_→(TTG)_9_	I	→Q				4.0			2.6		2.8		
*A9R*	259	(A)_8_→(A)_9_	FS												1.5
*C1L*	465	C→A	S	R→S						1.2					
*C2L*	168	C→T	N	none										1.5	
*D6L*	613	ATA→TCC	S	Y→G							1.9				
*D6L*	615	A→T	T					1.0					1.1		
*D6L*	616	+TCC	I	→G				8.8					7.4		
*D6L*	611	C→T	S	S→N				8.7					7.4		
*D6L*	606	G→A	N	none				8.5					7.1		
*E10R*	474	(T)_7_→(T)_8_	FS		2.9	2.0			1.7			2.2			
*E10R*	481	(T)_7_→(T)_8_	FS								2.6				
*E10R*	716	A→T	S	E→V						1.8					
*H3R*	28	A→G	S	N→D						1.3					
*J4L*	101	+GG	FS		1.1										
*J6R*	319	A→G	S	K→E											2.1
*L6L*	1084	(T)_10_→(T)_11_	FS		1.9										
*O4R*	1648	T→C	S	S→P										1.1	

Variant frequencies are shown in %; P0: crust; ^a^ Coding sequence (CDS; gene name according to CPXV GRI-90); ^b^ Position (Pos.) in gene; ^c^ Protein effect, S: Substitution, FS: Frame Shift, I: Insertion, D: Deletion, T: Truncation, N: None.

**Table 2:** Function of viral proteins changing in a cell type-specific manner during passaging.

Protein	Description ^a^	Function
HEp-2		
A34	EEV glycoprotein	membrane
E8	Cell-surface-binding protein	membrane
E13	Scaffold protein	membrane
B17	Soluble IFN-α/β receptor	immune evasion
C17	Complement control protein	immune evasion
D1/I5	Secreted chemokine-binding protein	immune evasion
Q1	Inhibitor of TNF-R and TLR signaling	immune evasion
C6	Uncharacterized protein	unknown
C10	Uncharacterized protein	unknown
F2	Uncharacterized protein	unknown
G16	Uncharacterized protein	unknown
A48	Cu-Zn superoxide dismutase-like protein	others
G2	dUTPase	others
H4	Glutaredoxin-2	others
A53	DNA ligase	DNA replication
Rat-2		
B4	EEV type-I membrane glycoprotein	membrane
A50	Uncharacterized protein	unknown
A52	Uncharacterized protein	unknown
B3	Uncharacterized protein	unknown
C14	Uncharacterized protein	unknown
B7	IFN-γ receptor-like protein	immune evasion
Q1	Inhibitor of TNF-R and TLR signaling	immune evasion
A53	DNA ligase	DNA replication

^a^ According to UniProt; nomenclature CPXV GRI-90.

**Table 3:** CPXV mature virion-associated host proteins that changed during passaging.

Gene name	Description	Enrichment in OPV IMV ^a^	Enrichment during Passaging ^b^				
			P1	P2	P3	P4	P5
HEp-2							
PFN-1	Profilin-1	35.1	1.3	0.5	−0.1	−0.6	−1.1
PGK1	Phosphoglycerate kinase 1	12.9	1.2	0.6	0.2	−1.3	−0.6
ARF1	ADP-ribosylation factor 1	12.8	1.4	0.4	−0.1	−1.0	−0.7
PDCD6	Programmed cell death protein 6	7.2	1.3	0.6	−0.5	−0.9	−0.6
XRCC5	X-ray repair cross-complementing protein 5	6.6	−1.1	−0.6	−0.2	0.5	1.4
PRDX6	Peroxiredoxin-6	4.3	1.5	0.4	−0.1	−1.0	−0.8
ATIC	Bifunctional purine biosynthesis protein	3.7	−1.2	0.1	−0.8	0.9	0.9
ACP1	Acid phosphatase 1	3.3	1.2	0.6	0.1	−1.1	−0.8
HSPA8	Heat shock cognate 71 kDa protein	2.4	1.0	0.5	0.6	−1.3	−0.7
CAD	CAD protein	2.4	−1.8	0.3	0.4	0.4	0.7
EIF3B	Eukaryotic translation initiation factor 3 subunit	2.1	−1.1	−0.8	0.2	0.3	1.4
MPG	DNA-3-methyladenine glycosylase	2.0	1.2	0.5	0.0	−1.2	−0.6
Rat-2							
Sqstm1	Sequestosome-1	8.3	0.5	1.2	0.0	−0.5	−1.2
Dstn	Destrin	7.6	1.1	−0.2	0.5	0.0	−1.4
Itpa	Inosine triphosphate pyrophosphatase	4.6	−1.4	−0.2	0.5	0.7	0.5
Phb2	Prohibitin-2	3.4	1.0	0.9	−0.6	−0.3	−1.0
Psmc5	26S protease regulatory subunit 8	3.4	−1.1	−0.8	0.4	0.7	0.9
Eif3b	Eukaryotic translation initiation factor 3 subunit B	2.1	−0.9	−1.0	0.3	0.8	0.8
Dnajc7	DnaJ homolog subfamily C member 7	2.1	0.8	0.6	−0.1	0.3	−1.6
Rab6a	Ras-related protein Rab-6A	2.0	0.5	1.2	−0.3	−0.3	−1.2

^a^ iBAQ ratio OPV IMV to HepG2 ≥ 2 according to [30]; ^b^ Log_2_ (normalized LFQ); P1–P5: passage 1–5.

## Supplementary Materials

Supplementary figures and tables are available online at www.mdpi.com/1999-4915/9/11/337/s1.
