# Supplementary material for: Combined Proteomics/Genomics Approach Reveals Proteomic Changes of Mature Virions as a Novel Poxvirus Adaptation Mechanism
Source: Viruses. 2017 Nov 10;9(11):337. doi: 10.3390/v9110337 (PMC5707544; doi:10.3390/v9110337)
Supplement: Supplementary file 1 [file viruses-09-00337-s001.pdf]

Supplementary Materials

Combined Proteomics/Genomics Approach Reveals Proteomic Changes of Mature Virions as a Novel Poxvirus Adaption Mechanism

Marica Grossegesse [1], Joerg Doellinger [1,2], Alona Tyshaieva [1], Lars Schaade [3] and Andreas Nitsche [1*]

Robert Koch Institute, Centre for Biological Threats and Special Pathogens (ZBS), 13353 Berlin, Germany

[1] Highly Pathogenic Viruses (ZBS 1); GrossegesseM@rki.de, TyshaievaA@rki.de, NitscheA@rki.de

[2] Proteomics and Spectroscopy (ZBS 6); DoellingerJ@rki.de

[3] Centre for Biological Threats and Special Pathogens (ZBS), SchaadeL@rki.de

* Correspondence: NitscheA@rki.de; Phone: +49 (0)30 18754 2313

Supplementary figures

Figure S1. Reference profiles of adaption-specific viral proteins.

Figure S2. CPE documentation during CPXV crust passaging.

Figure S3. Real-time cell analysis of crust and passages in HEp-2 and Rat-2 cells.

Figure S4. Coverage across CPXV genome.

Figure S5. Heatmap visualization of variant frequencies shared in HEp-2 and Rat-2 cells.

Figure S6. Linear correlation of virus protein intensities in IMV particles during passaging.

Figure S7. Principal component analysis of host CPXV IMV proteins during passaging.

Figure S8. Heatmap visualization of host proteins changing during passaging.

Figure S9. Plaque size during passaging.

Supplementary tables

Table S1. Determination of the GE-to-PFU ratio.

Table S2. Parameters LC-MS/MS.

Table S3. Parameters MaxQuant.

Table S4. Overview of poxvirus reads and coverage.

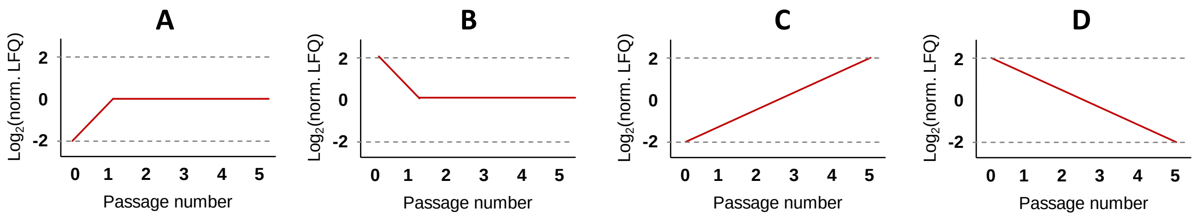

Figure S1. Reference profiles of adaption-specific viral proteins. (**A**,**B**) Reference profiles for viral proteins showing adaption upon host switch. (**C**,**D**) Reference profiles for viral proteins showing a continuous adaption during passaging.

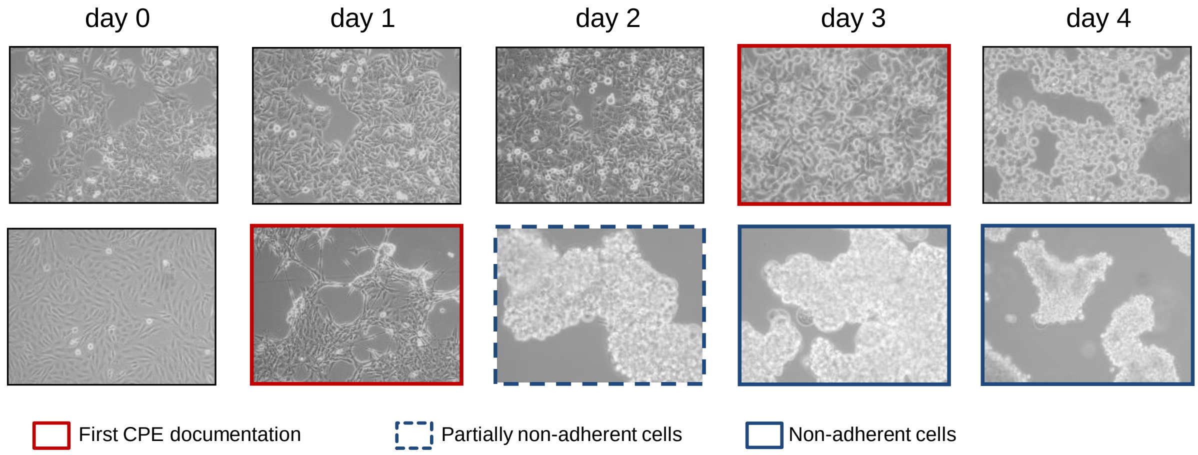

Figure S2. CPE documentation during CPXV crust passaging. HEp-2 and Rat-2 cells were infected with purified CPXV isolated from a rat crust (day 0) at an MOI of 0.1 and the cytopathic effect (CPE) was documented daily until harvesting (day 4).

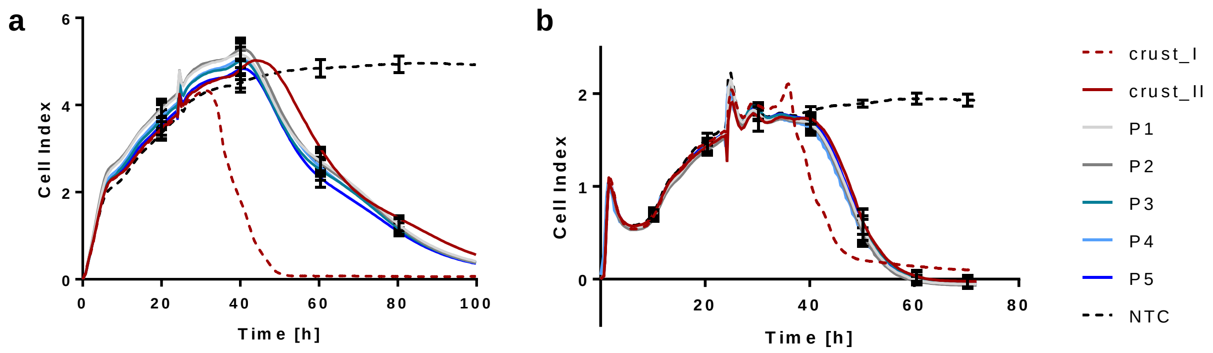

Figure S3. Real-time cell analysis of crust and passages in HEp-2 and Rat-2 cells. Repetition of the experiment. (**a**) HEp-2 cells; (**b**) Rat-2 cells. Shown are mean values ± SD of biological triplicates of passages measured in technical quadruplicates (n=12) or four (HEp-2) or five (Rat-2) technical replicates of the crust. One replicate of the crust in each cell line shows faster cell detachment (crust_I).

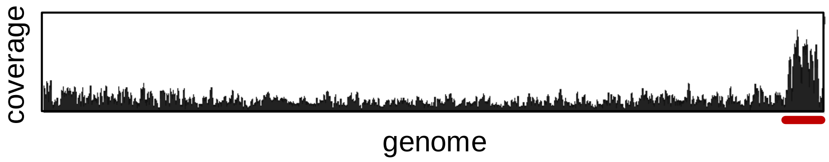

Figure S4. Coverage across CPXV genome. Duplicated genes display increased coverage (red bar).

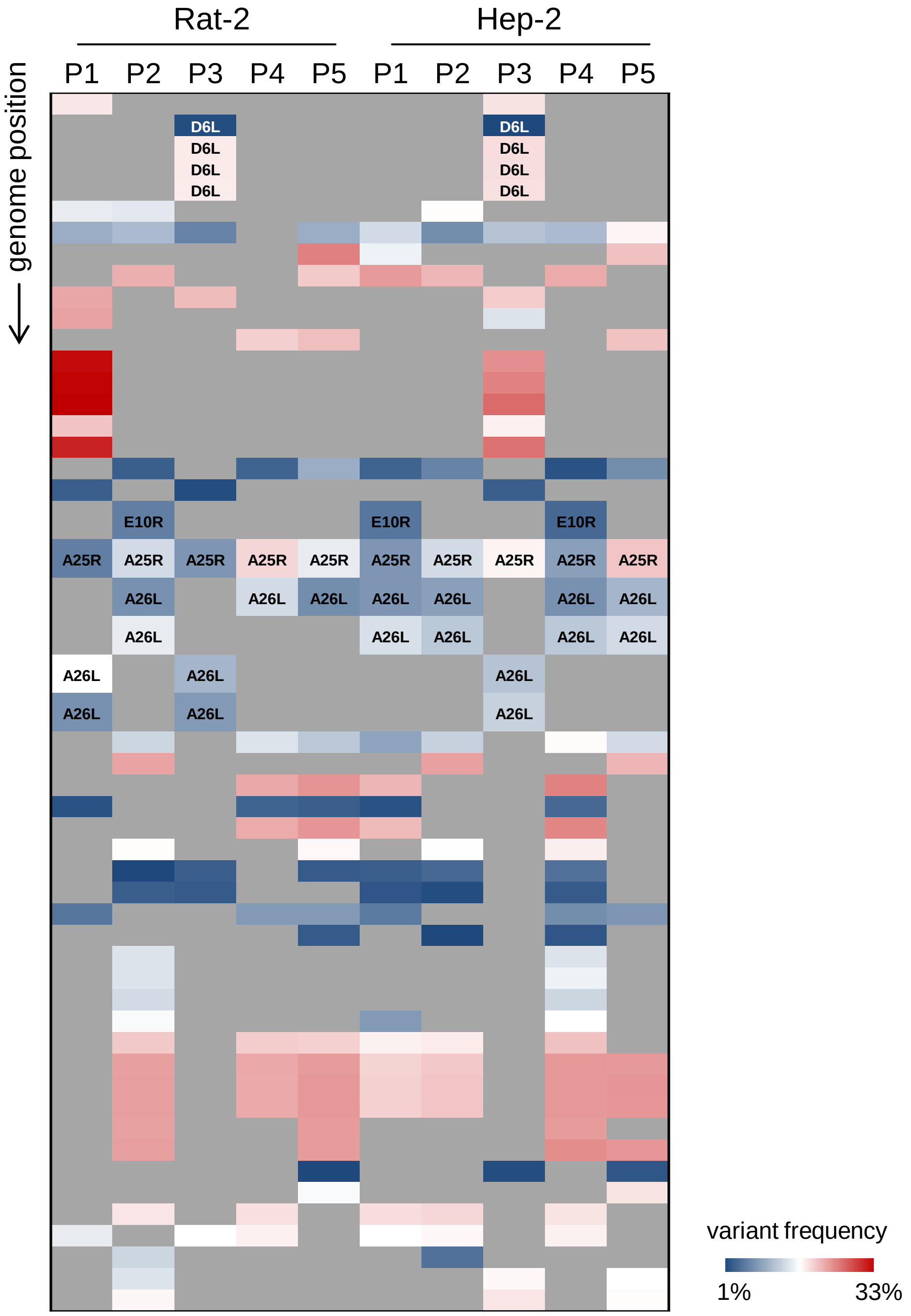

Figure S5. Heatmap visualization of variant frequencies shared in HEp-2 and Rat-2 cells. The frequency of 54 variants detected in both cell lines is shown according to the genome position. Frequencies are comparable, but

variants appeared mostly in different passages. If no gene name is shown variants localize in non-coding regions. Grey: variant not detected.

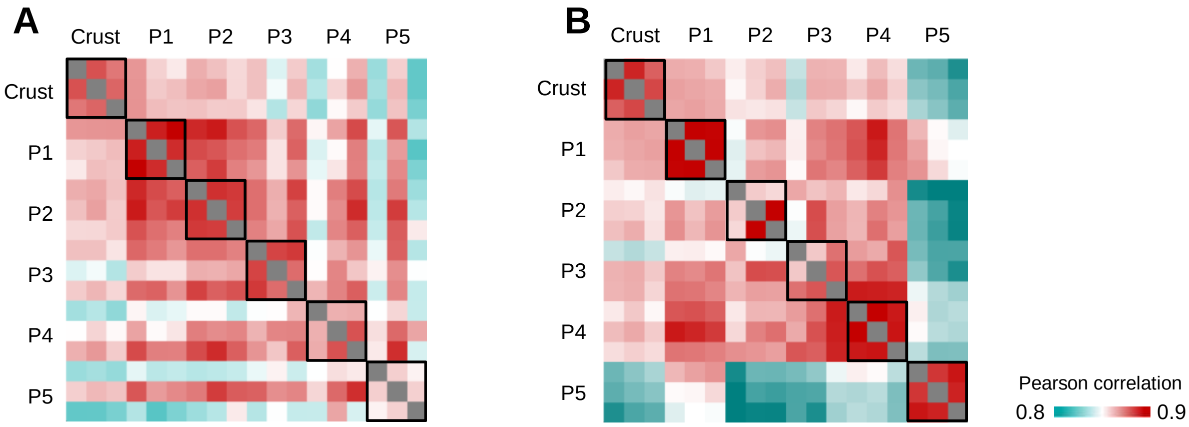

Figure S6. Linear correlation of virus protein intensities in IMV particles during passaging. Shown is the Pearson correlation of normalized valid viral LFQ intensities without missing values of crust and cell culture passaged virus (P1-P5) in (**A**) HEp-2 and (**B**) Rat-2 cells. P1-5=passages 1-5.

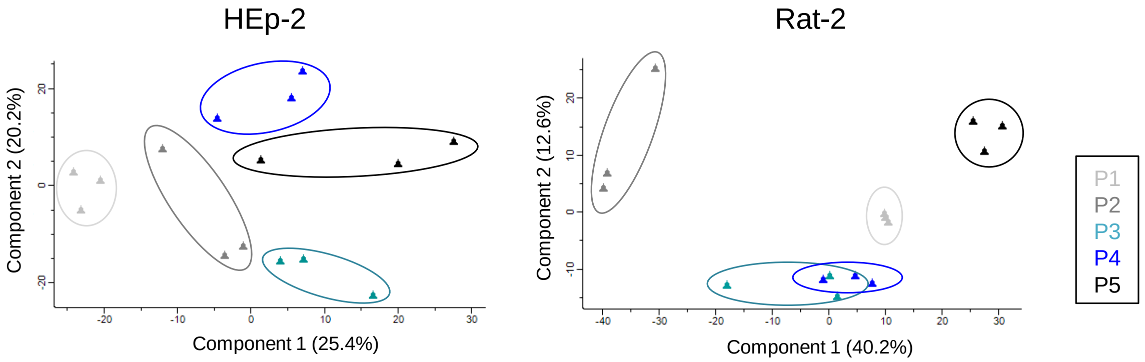

Figure S7. Principal component analysis of host CPXV IMV proteins during passaging. Normalized LFQ intensities of virus proteins without missing values were used for principal component analysis, revealing separability of host cell proteins associated with CPXV IMV particles during passaging in HEp-2 and Rat-2 cells. P1-P5=passage 1-5.

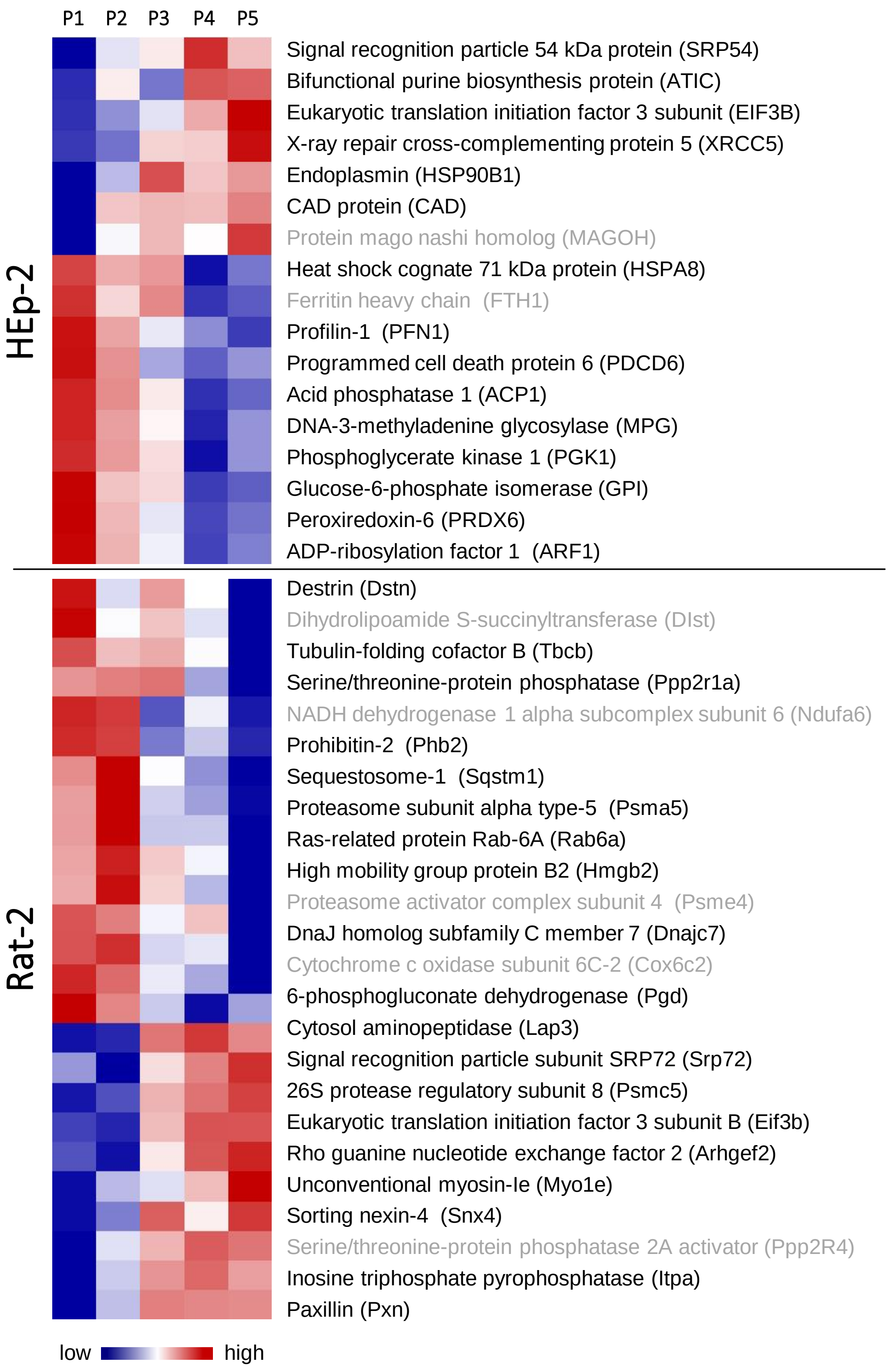

Figure S8. Heatmap visualization of host proteins changing during passaging. CPXV were passaged in HEp-2 and Rat-2 cells (P1-P5=passage 1-5) and analyzed for virion-associated host proteins. Shown are mean values of three biological replicates of z-score normalized protein intensities (ANOVA significant). Ribosomal proteins and histones were removed. Proteins written in black have been previously identified in highly pure OPV IMV preparations [1].

1. Doellinger, J.; Schaade, L.; Nitsche, A. Comparison of the cowpox virus and vaccinia virus mature virion

proteome: Analysis of the species- and strain-specific proteome. *PLoS One* **2015**, *10*, e0141527.

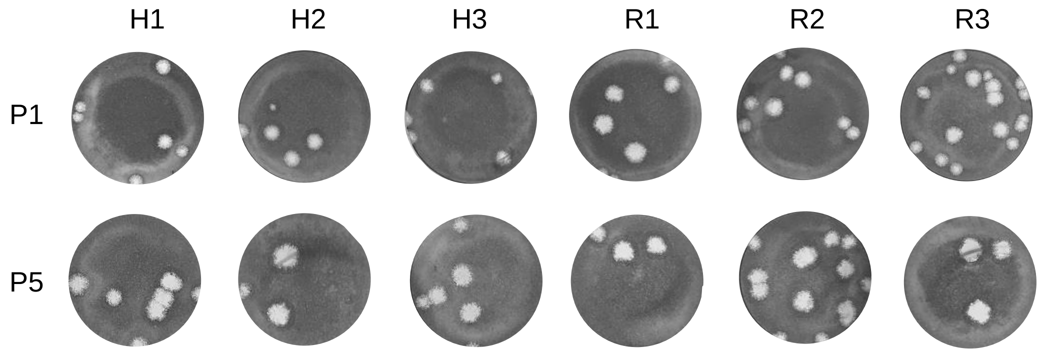

Figure S9. Plaque size during passaging. Letter indicate cell line (H=HEp-2, R=Rat-2) and replicate number (1-3); P1= first passage; P5= fifth passage.

Table S1. Determination of the GE-to-PFU ratio.

Sample	GE	PFU	GE/PFU ratio	Mean	SD
HEp-2, replicate 1	2.48E+10	3.50E+08	70.8	**82**	9
HEp-2, replicate 2	2.61E+10	3.33E+08	78.3		
HEp-2, replicate 3	2.47E+10	2.83E+08	87.2		
HEp-2, replicate 4	3.62E+10	4.00E+08	90.6		
Rat-2, replicate 1	2.50E+09	1.75E+08	14.3	**17**	8
Rat-2, replicate 2	3.28E+09	1.17E+08	28.2		
Rat-2, replicate 3	2.09E+09	2.17E+08	9.6		
Rat-2, replicate 4	4.00E+09	2.33E+08	17.1		

Table S2. Parameters LC-MS/MS.

**nLC separation**	
Analytical column	Reprosil-Pur 120 C18-AQ, 2.4 µm, 300 mm x 75 µm
Emitter tip	Stainless steel emitter, O.D. 150 µm, I.D. 30 µm
Solvent A	0.1 % FA, 3 % DMSO in H_2O
Solvent B	0.1 % FA, 3 % DMSO in ACN
Flow rate	225 nl/min
Gradient	0-29 % B in 4 h
Sample load	2 µg in 4 µl in 0.1 % FA
**LTQ Orbitrap**	
Top N iontrap	7
Resolution orbitrap	30000
Scan rate ion trap	normal
Ion charge	+2, +3
AGC MS target value	1.00E+06
AGC MS^2 target value	5.00E+03
MS^2 threshold	5.00E+02
Mass range	400-1400 m/z
Normalized collision energy for CID	35 %
Max. ion accumulation time MS	500 ms
Max. ion accumulation time MS^2	100 ms
Exclusion duration	120 s
Spray voltage	2.0 kV
Capillary temperature	275 °C

Table S3. Parameters MaxQuant.

RAW files	3× crust<br>(technical replicates)<br>15× HEp-2 P1-P5<br>15× Rat-2 P1-P5
Databases	Human, rat, CPXV_crust Hei, contaminant
Digestion mode, enzyme	Specific, trypsin/P
Max missed cleavages	2
Variable modifications	Oxidation (M), acetylation (protein N-term), diGly(K)
Fixed modification	Carbamidomethyl (C)
Mass tolerance parent ions	6 ppm
Mass tolerance fragment ions	0.5 Da
Peptide FDR	0.05
Protein FDR	0.01
Site decoy fraction	0.01
LFQ min. ratio count	1
Match between runs	ON
Match time window [min]	2.5
Alignment time window [min]	20
Peptides for quantification	Unique + razor
Use only unmodified peptides for quantification	OFF

Table S4. Overview of poxvirus reads and coverage.

Sample[a]	Pox reads[b]	Coverage[c]	Genome size	Sample[a]	Pox reads[b]	Coverage[c]	Genome size
HP1	3.27E+06	2432.6	216,329	RP1	1.74E+06	1338.4	209,458
HP2	4.28E+06	3596.3	216,478	RP2	6.80E+06	5624.3	216,476
HP3	3.15E+06	3233.1	216,230	RP3	6.94E+06	6114.5	216,481
HP4	3.01E+06	3014.3	216,626	RP4	8.54E+05	722.1	216,479
HP5	1.13E+06	891.3	216,476	RP5	7.97E+05	793.4	216,899
Mean	2.97E+06	2633.5		Mean	3.43E+06	2918.54	
crust	1.30E+06	1290.3	216,458				

[a]H=HEp-2; R=Rat-2; P=passage; [b]Trimmed reads after quality control; [c]Mean across viral genome
